# Aggregation of TDP-43 fragments triggers cellular aberrant function: implication in neurodegeneration

**DOI:** 10.1186/1750-1326-7-S1-O1

**Published:** 2012-02-07

**Authors:** Meixia Che, Leilei Jiang, Chenjie Zhou, Yajun Jiang, Hongyu Hu

**Affiliations:** 1 State Key Laboratory of Molecular Biology, Institute of Biochemistry and Cell Biology, Shanghai Institutes for Biological Sciences, Chinese Academy of Sciences, Shanghai, China

## 

TAR DNA binding protein of 43 kDa (TDP-43) is a nuclear factor functioning in RNA processing. It was also reported that TDP-43 aggregates are deposited in motor neurons implicated in the pathogenesis of amyotrophic lateral sclerosis (ALS) and frontotemporal lobar degeneration with ubiquitin (FTLD-U). To understand the mechanism underlying the inclusion body formation and possible functional alteration of TDP-43, we studied TDP-43 and its fragments and their effects on RNA processing *in vitro* and in cell models. We have identified a hydrophobic sequence in the C-terminus that is critical for the TDP-43 aggregation and inclusion formation. The synthetic peptide with this sequence forms an α-helical structure in solution but easily transforms into β-sheet structure. The inclusions formed by the C-terminal 35-kDa fragment (TDP-35) can recruit full-length TDP-43 to cytoplasmic deposition from functionally nuclear localization. TDP-35, rather than TDP-43 and the C-terminal 25-kDa fragment, is prone to aggregation *in vitro*, and it can further serve as a seed to facilitate aggregation of full-length TDP-43. This suggests that fragmentation of TDP-43 leads to cellular redistribution, inclusion body formation, and altered RNA processing, which are implicated in the molecular pathogenesis of ALS and FTLD.

